# Long-Term Outcomes of Patients Undergoing Conversion Surgery After Induction Chemotherapy: Turkish Oncology Group Study

**DOI:** 10.3390/medicina61050776

**Published:** 2025-04-22

**Authors:** Furkan Ceylan, Selin Aktürk Esen, Olçun Ümit Ünal, Ferit Aslan, İlknur Deliktaş Onur, Öztürk Ateş, Erkut Demirciler, İlkay Tuğba Ünek, Ahmet Gülmez, Esra Özen Engin, Semra Taş, Gamze Gököz Doğu, Melih Şimşek, Hacı Mehmet Türk, Ali İnal, Gökhan Şahin, Haydar Çağatay Yüksel, Ateş Kutay Tenekeci, Mutlu Hızal, Mehmet Ali Nahit Şendur, Doğan Uncu

**Affiliations:** 1 Department of Medical Oncology, Ankara Bilkent City Hospital, Ankara 06800, Turkey; drselin16@hotmail.com (S.A.E.); drmutluhizal@hotmail.com (M.H.); masendur@yahoo.com.tr (M.A.N.Ş.); doganuncu@yahoo.com (D.U.); 2 Department of Medical Oncology, UHS Izmir Faculty of Medicine, Izmir 35020, Turkey; drolcun@hotmail.com; 3 Department of Medical Oncology, Ankara Batıkent Medical Park Hospital, Ankara 06680, Turkey; feritferhat21@gmail.com; 4 Department of Medical Oncology, A.Y. Ankara Oncology Training and Research Hospital, Ankara 06200, Turkey; ilknurdeliktas382@gmail.com (İ.D.O.); dr.ozturkates@gmail.com (Ö.A.); 5 Department of Medical Oncology, Dokuz Eylül University, Izmir 35390, Turkey; erkutdemirciler@gmail.com (E.D.); ilkaytugbaunek@gmail.com (İ.T.Ü.); 6 Department of Medical Oncology, Adana City Hospital, Adana 01230, Turkey; doktor.ahmetgulmez@gmail.com; 7 Department of Medical Oncology, Sakarya Training and Research Hospital, Sakarya 54100, Turkey; dresraozen@gmail.com; 8 Department of Medical Oncology, Pamukkale University, Denizli 20070, Turkey; semratasdr@gmail.com (S.T.); ggd2882@gmail.com (G.G.D.); 9 Department of Medical Oncology, Bezmialem University, Istanbul 34093, Turkey; mdmelih@gmail.com (M.Ş.); hmturk@bezmialem.edu.tr (H.M.T.); 10 Department of Medical Oncology, Mersin City Hospital, Mersin 33330, Turkey; dr.ainal@gmail.com; 11 Department of Medical Oncology, Ege University, Izmir 35100, Turkey; gkhn7sn@gmail.com (G.Ş.); haydar.cagatay.yuksel@ege.edu.tr (H.Ç.Y.); 12 School of Medicine, Hacettepe University, Ankara 06100, Turkey; atesktenekeci@gmail.com; 13 Department of Medical Oncology, Ankara Yıldırım Beyazıt University, Çankaya 06800, Turkey

**Keywords:** anti-EGFR therapy, anti-VEGF therapy, conversion surgery, induction chemotherapy, liver metastasis, metastatic colorectal cancer

## Abstract

*Background and Objectives*: Conversion surgery for liver metastatic colorectal cancer (mCRC) has been associated with prolonged survival. This study aimed to evaluate the efficacy and safety of integrating biological therapies with fluorouracil-based induction chemotherapy in patients with isolated liver mCRC who subsequently underwent curative resection of both the primary tumor and liver metastases. *Materials and Methods:* This multicenter, retrospective study, conducted by the Turkish Oncology Group (TOG), included 116 patients from 11 tertiary centers who underwent conversion surgery following induction chemotherapy between 2009 and 2024. *Results:* The median age was 57 years, with 62% male patients. The median follow-up period was 55.3 months. The median progression-free survival (PFS) and overall survival (OS) were 21.1 and 53.7 months, respectively. No significant differences in PFS or OS were observed based on biological therapy use or tumor localization. Among patients with RAS/RAF wild-type tumors, PFS and OS were comparable between those receiving Anti-EGFR and Anti-VEGF therapy. In RAS/RAF mutant tumors, the addition of Anti-VEGF therapy did not confer a survival benefit. Factors associated with shorter PFS included advanced tumor stage (ypT3-T4), lymph node metastasis, and multiple metastases, while shorter OS was linked to advanced tumor stage and lack of objective response. *Conclusions:* Surgical resection plays a pivotal role in improving survival outcomes in patients with potentially resectable liver mCRC. Optimizing induction chemotherapy regimens may enhance conversion rates and prolong long-term survival. Further studies are needed to refine treatment selection based on tumor localization, mutation status, and molecular biomarkers.

## 1. Introduction

Colorectal cancer (CRC) is the third most commonly diagnosed malignancy worldwide in both men and women, accounting for approximately 10% of all cancer-related deaths [1]. At the time of diagnosis, approximately 20% of patients are present with metastatic disease. According to GLOBOCAN 2022 data, in Turkey, there were 21,718 new cases of colorectal cancer (9%), making it the third most common cancer. Additionally, 11,700 cancer-related deaths (9%) were reported [2]. While advancements in systemic therapies have extended overall survival (OS) beyond 30 months, further gains can be achieved through an integrated approach that includes systemic chemotherapy, targeted therapies, and local treatment modalities.

The liver is the predominant site of metastasis in CRC, with synchronous liver metastases observed in 15–25% of cases [3]. In Turkey, the rate of synchronous metastasis is similar to that observed worldwide [4]. Effective management of isolated liver metastases is essential for improving long-term survival. Among local treatment strategies, surgical resection remains the most effective approach, offering the potential for prolonged disease control and even cure [5]. However, the optimal sequencing of chemotherapy and surgery remains a subject of debate, necessitating individualized decision-making by multidisciplinary tumor boards [6]. Several factors complicate the treatment paradigm for resectable metastatic CRC (mCRC), including interpatient variability, the underrepresentation of elderly and comorbid patients in clinical trials, the subjective nature of resectability assessments, and institutional differences in treatment strategies. In metastatic colorectal cancer, it is well known that the addition of biologic therapies to fluorouracil-based chemotherapy enhances the depth of response [7,8]. Therefore, their use in induction chemotherapy is applicable in potentially resectable mCRC cases [9]. However, the effect of adding biologic agents to induction chemotherapy on long-term outcomes in patients who undergo surgery remains a subject of ongoing interest.

This study aims to assess the efficacy and safety of incorporating targeted biological therapies into fluorouracil-based induction chemotherapy in patients with isolated liver mCRC who underwent curative resection of both the primary tumor and liver metastases following systemic therapy.

## 2. Material and Methods

This multicenter, retrospective study was conducted by the Turkish Oncology Study Group (TOG). It included 116 patients with isolated liver metastatic colorectal cancer (mCRC) diagnosed between January 2009 and January 2024 across 11 tertiary oncology centers in Turkey. Treatment decisions were determined by multidisciplinary tumor boards, comprising specialists in medical oncology, radiation oncology, gastroenterological surgery, and radiology. The study was approved by the Ankara Bilkent City Hospital Ethics Committee (E1/4555/2023) and conducted in accordance with the Declaration of Helsinki.

The study enrolled patients aged 18 years or older with histologically confirmed colorectal adenocarcinoma with isolated liver metastases, initially classified as unresectable by a multidisciplinary tumor board. Following induction chemotherapy with or without targeted therapy, these patients underwent a subsequent re-evaluation by the same multidisciplinary team and were deemed eligible for curative-intent conversion surgery. All patients underwent simultaneous hepatic metastasectomy and primary tumor resection during the surgical intervention.

Exclusion criteria included initially resectable liver metastases, the presence of extrahepatic metastatic disease, or primary tumor resection at the time of diagnosis due to obstruction or perforation. Patients who did not undergo curative-intent liver resection following systemic therapy were also excluded from the study.

Clinicopathologic characteristics, radiologic findings, treatments, and adverse effects were retrospectively analyzed from patient records. Extended RAS and RAF mutations were assessed using polymerase chain reaction (PCR), while DNA mismatch repair (MMR) status was evaluated via immunohistochemistry. Tumor regression was graded according to the modified Ryan classification, where grade 0 indicates the absence of viable cancer cells, grade 1 represents single or rare small clusters of cancer cells, grade 2 denotes tumor regression with multiple tumor cell clusters, and grade 3 reflects the absence of tumor regression [10,11]. Radiologic treatment response was assessed according to Response Evaluation Criteria in Solid Tumors (RECIST 1.1), with evaluations performed by radiologists at each participating center [12]. The primary endpoint was progression-free survival (PFS), defined as the time from diagnosis to recurrence or death. Overall survival (OS), defined as the time from diagnosis to death from any cause or last follow-up, served as the secondary endpoint.

### 2.1. Treatment Regimens

The treatment regimen administered in this study included various chemotherapy protocols, either alone or in combination with biologic therapies. Patients received FOLFOX (fluorouracil 400 mg bolus, 2400 mg/m^2^ continuous infusion, leucovorin 400 mg/m^2^, oxaliplatin 85 mg/m^2^), FOLFIRI (fluorouracil 400 mg bolus, 2400 mg/m^2^ continuous infusion, leucovorin 400 mg/m^2^, irinotecan 180 mg/m^2^), CAPOX (capecitabine 2000 mg/m^2^, oxaliplatin 130 mg/m^2^), or FOLFOXIRI (fluorouracil 400 mg bolus, 2400 mg/m^2^ continuous infusion, leucovorin 400 mg/m^2^, oxaliplatin 85 mg/m^2^, irinotecan 180 mg/m^2^). Biologic therapies included bevacizumab (5 mg/kg), panitumumab (6 mg/kg), or cetuximab (500 mg/m^2^), administered alongside chemotherapy based on molecular profiling and clinical indications.

### 2.2. Statistics

Statistical analyses were conducted using SPSS version 26.0 (Chicago, IL, USA). Descriptive statistics were presented as mean ± standard deviation or median with interquartile range, depending on data distribution. Comparisons of numerical variables were performed using the Student’s *t*-test for normally distributed data and the Mann–Whitney U test for non-normally distributed data. Categorical variables were analyzed using the chi-square test. Progression-free survival (PFS) and overall survival (OS) were assessed using the Kaplan–Meier method and compared between groups with the log-rank test. Cox regression analysis was utilized to identify independent prognostic factors for survival. Follow-up duration was determined using the reverse Kaplan–Meier method. The receiver operating characteristic (ROC) curve was applied to evaluate the largest liver metastasis size. A *p*-value < 0.05 was considered statistically significant.

## 3. Results

### 3.1. Baseline Characteristics

A total of 116 patients were included in the study, with a median age of 57 years (IQR: 42–72), and 62% were male. The median follow-up duration was 55.3 months, with median PFS and OS of 21.1 and 53.7 months, respectively. Tumor localization was distributed as follows: 24% in the right colon (*n* = 28), 34% in the left colon (*n* = 40), and 42% in the rectum (*n* = 48). Among the treatment regimens, 14 patients (13%) received doublet chemotherapy alone, while 56 patients (52%) received anti-VEGF therapy, and 37 patients (35%) received anti-EGFR therapy. Clinicopathologic characteristics and treatment details are presented in Table 1.

When patients were categorized by chemotherapy regimen, baseline characteristics were largely comparable, except for a higher female-to-male ratio among those receiving doublet chemotherapy alone compared to those receiving doublet + anti-EGFR or doublet + anti-VEGF therapies (71%, 32%, and 34%, respectively; *p* = 0.024). Additionally, patients treated with doublet + anti-EGFR received significantly more chemotherapy cycles compared to those receiving doublet + anti-VEGF or doublet chemotherapy alone (11.6, 8, and 7 cycles, respectively; *p* = 0.004). The incidence of grade 3/4 adverse events was significantly higher in the doublet + anti-EGFR group than in the doublet + anti-VEGF and doublet chemotherapy groups (*p* < 0.001) (Supplementary Table S1).

When analyzed based on tumor localization, patient and tumor characteristics were largely similar. However, in left-sided colon tumors, liver metastases were less frequently confined to the same segment compared to right-sided colon and rectal tumors (*p* = 0.044) (Table 1). A summary of treatments based on tumor localization is provided in Supplementary Table S2.

### 3.2. Progression-Free Survival

**Figure 1:**
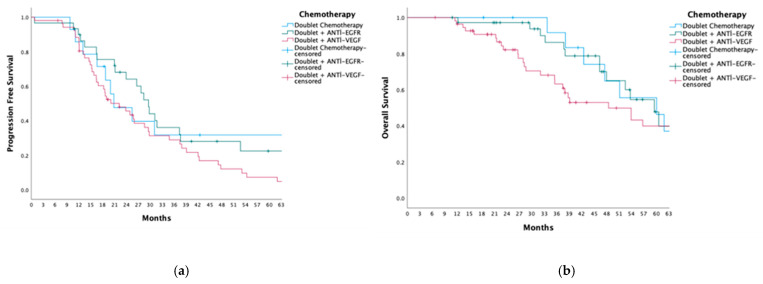
Kaplan–Meier Curves for Progression-Free Survival and Overall Survival Based on Chemotherapy Regimens. (**a**) Progression-Free Survival (PFS): Kaplan–Meier survival estimates for patients receiving doublet chemotherapy, doublet chemotherapy plus anti-EGFR therapy, and doublet chemotherapy plus anti-VEGF therapy. The median PFS for doublet chemotherapy, doublet + anti-EGFR, and doublet + anti-VEGF groups was 20.8 months, 29.6 months, and 22.2 months, respectively (*p* = 0.095). (**b**) Overall Survival (OS): Kaplan–Meier survival estimates for patients treated with doublet chemotherapy, doublet + anti-EGFR, and doublet + anti-VEGF therapies. The median OS for these groups was 60.1 months, 59.5 months, and 48.5 months, respectively (*p* = 0.199). Censored data points are indicated by tick marks on the curves.

Progression-free survival (PFS) was comparable among patients receiving doublet chemotherapy, doublet + anti-EGFR, and doublet + anti-VEGF therapy, with median PFS values of 20.8, 29.6, and 22.2 months, respectively (*p* = 0.095) (Figure 1). Similarly, there was no significant difference in PFS based on tumor location, as patients with right-sided colon tumors had a median PFS of 18.6 months, whereas those with left-sided colon and rectal tumors had a median PFS of 22.8 months (*p* = 0.876).

**Figure 2:**
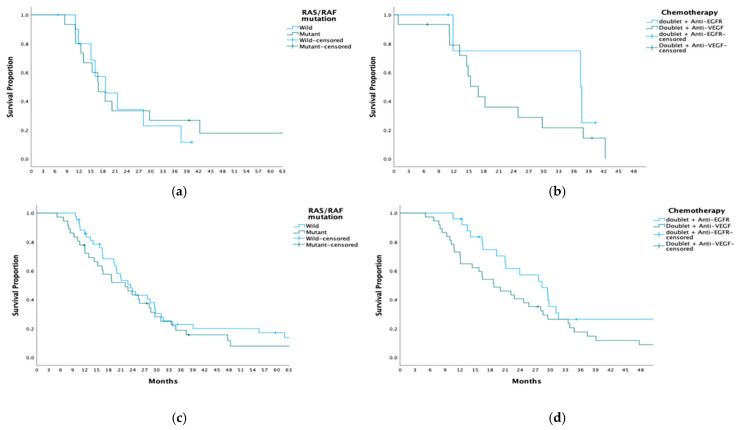
Progression-Free Survival (PFS) by Tumor Location and Therapy. (**a**) Right colon tumors: Comparison of RAS/RAF wild-type vs. RAS/RAF mutant groups (16.9 vs. 18.7 months, *p* = 0.808). (**b**) Right colon tumors: PFS in patients receiving doublet therapy with anti-EGFR vs. doublet therapy with anti-VEGF (37.6 vs. 16.1 months, *p* = 0.275). (**c**) Left colon tumors: Comparison of RAS/RAF wild-type vs. RAS/RAF mutant groups (22.1 vs. 23.5 months, *p* = 0.395). (**d**) Left colon tumors: PFS in patients receiving doublet therapy with anti-EGFR vs. doublet therapy with anti-VEGF (28.3 vs. 18.7 months, *p* = 0.051).

In the subgroup analysis of right-sided colon tumors, median PFS was similar between patients with and without RAS/RAF mutations (16.9 vs. 18.7 months, *p* = 0.808). Among these patients, PFS did not differ significantly between those treated with anti-EGFR plus doublet therapy and those receiving anti-VEGF plus doublet therapy (37.6 vs. 16.1 months, *p* = 0.275) (Figure 2). In RAS/RAF wild-type tumors, PFS was numerically longer in the anti-EGFR group compared to the anti-VEGF group, although this difference did not reach statistical significance (37.6 vs. 15.0 months, *p* = 0.059). In patients with RAS/RAF mutant right-sided colon tumors, PFS was similar between those treated with anti-VEGF plus doublet therapy and anti-VEGF plus triplet therapy (16.7 vs. 11.2 months, *p* = 0.246) (Supplementary Figure S1).

In left-sided colon and rectal tumors, median PFS was comparable between patients with and without RAS/RAF mutations (23.5 vs. 22.1 months, *p* = 0.395). Among these patients, PFS was numerically longer in those receiving anti-EGFR plus doublet therapy compared to anti-VEGF therapy, though the difference was not statistically significant (28.3 vs. 18.7 months, *p* = 0.051) (Figure 1). In RAS/RAF wild-type tumors, PFS was similar between anti-EGFR plus doublet therapy and anti-VEGF plus doublet therapy (28.3 vs. 20.0 months, *p* = 0.173). In RAS/RAF mutant tumors, PFS was comparable across fluorouracil-based doublet therapy, fluorouracil-based triplet therapy, and triplet therapy plus anti-VEGF (22.8, 23.9, and 18.6 months, respectively, *p* = 0.501) (Supplementary Figure S1).

Multivariate analysis identified advanced tumor T stage (ypT3-T4 vs. ypT1-T2: HR: 2.33, 95% CI, *p* = 0.072), lymph node metastasis (N+ vs. N0: HR: 1.74, 95% CI, *p* = 0.053), and the presence of more than three metastases (HR: 2.65, 95% CI, *p* = 0.002) as factors significantly associated with shorter PFS. Detailed univariate and multivariate analyses for PFS are presented in Table 2.

### 3.3. Overall Survival

Overall survival (OS) was comparable among patients receiving doublet chemotherapy, doublet + anti-EGFR therapy, and doublet + anti-VEGF therapy, with median OS values of 60.1, 59.5, and 48.5 months, respectively (*p* = 0.199). Similarly, OS did not significantly differ by tumor location, with patients having right-sided colon tumors exhibiting a median OS of 53.9 months, while those with left-sided colon and rectal tumors had a median OS of 53.7 months (*p* = 0.580).

**Figure 3:**
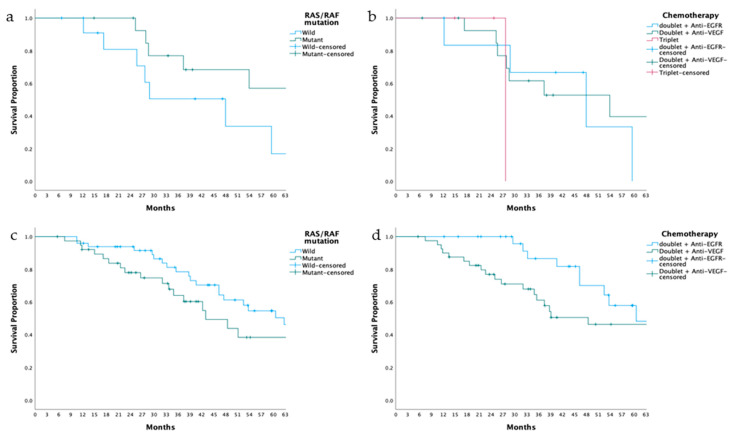
Overall Survival (OS) by Tumor Location and Therapy. (**a**) Right colon tumors: Comparison of RAS/RAF wild-type vs. RAS/RAF mutant groups (48.0 vs. 63.2 months, *p* = 0.127). (**b**) Right colon tumors: OS in patients receiving doublet therapy with anti-EGFR vs. doublet therapy with anti-VEGF vs. triplet therapy (48.0 vs. 53.9 vs. 27.7 months, *p* = 0.583). (**c**) Left colon tumors: Comparison of RAS/RAF wild-type vs. RAS/RAF mutant groups (62.7 vs. 43.0 months, *p* = 0.184). (**d**) Left colon tumors: OS in patients receiving doublet therapy with anti-EGFR vs. doublet therapy with anti-VEGF (60.6 vs. 48.5 months, *p* = 0.055).

In the subgroup analysis of right-sided colon tumors, OS was similar between patients with and without RAS/RAF mutations (63.2 vs. 48.0 months, *p* = 0.127). Among these patients, OS was numerically longer in those receiving anti-VEGF plus doublet therapy compared to doublet + anti-EGFR therapy or triplet therapy (53.9 vs. 48.0 and 27.7 months, *p* = 0.583), though this difference was not statistically significant (Figure 3). In RAS/RAF wild-type patients, OS was numerically longer in the anti-EGFR group compared to the anti-VEGF group or triplet therapy (48.0 vs. 25.6 and 27.7 months, *p* = 0.711), though the difference did not reach statistical significance. In RAS/RAF mutant patients, OS was similar between those receiving doublet therapy alone and those receiving doublet therapy plus anti-VEGF (not estimable vs. 53.9 months, *p* = 0.172) (Supplementary Figure S2).

For left-sided colon and rectal tumors, OS was similar between RAS/RAF wild-type and mutant patients (43.0 vs. 62.7 months, *p* = 0.184). Patients treated with doublet chemotherapy plus anti-EGFR therapy had numerically longer OS compared to those receiving doublet plus anti-VEGF (60.6 vs. 48.5 months, *p* = 0.055), but the difference was not statistically significant (Figure 2). In RAS/RAF wild-type tumors, OS was similar between patients receiving doublet + anti-VEGF and those treated with doublet + anti-EGFR therapy (69.0 vs. 60.6 months, *p* = 0.230). In RAS/RAF mutant tumors, OS was comparable across doublet therapy, doublet plus anti-VEGF, and triplet therapy (51.1, 48.5, and 43.0 months, *p* = 0.798) (Supplementary Figure S2).

Multivariate analysis identified advanced tumor T stage (ypT3-T4 vs. ypT1-T2: HR: 12.2, 95% CI, *p* = 0.040) and lack of objective response (HR: 8.41, 95% CI, *p* = 0.023) as significant predictors of shorter OS. Detailed univariate and multivariate analyses for OS are presented in Table 3.

### 3.4. Radiographic Response and Safety

In right-sided colon tumors, the overall response rate (ORR) was comparable across treatment groups: 100% in patients receiving doublet chemotherapy, 86% in those treated with anti-EGFR therapy, 87% in the anti-VEGF group, and 100% in those receiving triplet therapy. Similarly, in left-sided colon and rectal tumors, ORR was 100% in patients treated with doublet chemotherapy, anti-EGFR therapy, and triplet therapy, while it was 85% in the anti-VEGF group.

The incidence of grade 3/4 adverse events was significantly higher in patients receiving anti-EGFR therapy compared to those treated with anti-VEGF or triplet therapy (38%, 7%, and 7%, respectively; *p* < 0.001). A total of 19 patients experienced grade ≥ 3 adverse events. Hematological toxicity was the most common, affecting 11 patients, with neutropenia in 10 patients, thrombocytopenia in one patient, and anemia in two patients. Additionally, one patient developed mucositis, three experienced diarrhea, one developed acute kidney injury, four had skin toxicity, and two reported fatigue.

## 4. Discussion

In this multicenter, retrospective study utilizing real-world data from Turkey, we evaluated the efficacy and safety of adding anti-EGFR or anti-VEGF therapy to fluorouracil-based treatment in patients with isolated liver metastatic colorectal cancer (mCRC) who underwent conversion surgery. The median OS and PFS for the entire cohort were 53.7 and 21.1 months, respectively. Survival outcomes (PFS and OS) did not significantly differ based on RAS/RAF mutation status, tumor localization, or treatment regimen. Among patients with RAS/RAF wild-type tumors, PFS and OS were comparable between those receiving anti-EGFR and anti-VEGF therapy. In RAS/RAF mutant tumors, the addition of anti-VEGF therapy did not confer a PFS or OS advantage. Systemic treatment strategies for metastatic CRC are primarily determined based on tumor localization, microsatellite instability (MSI) status, RAS/RAF mutation status, and patient characteristics. In potentially resectable liver metastatic colorectal cancer, a similar systemic treatment is administered with the goal of reducing tumor size to facilitate conversion surgery. Although the addition of biological agents to fluorouracil-based chemotherapy has been shown to enhance tumor response depth, its impact on resectability rates and recurrence prevention remains uncertain because data on this subset are derived from the fraction of patients who ultimately undergo surgery, representing a limited group among all mCRC patients receiving systemic therapy. The presence and depth of objective response are particularly critical in this patient population, as only 7% of patients with initially unresectable isolated liver mCRC can ultimately undergo curative surgery following induction chemotherapy [13].

Induction chemotherapy not only facilitates conversion surgery in initially unresectable patients but also reduces the risk of recurrence by targeting micrometastatic disease [14].

In studies involving metastatic colorectal cancer (mCRC) patients who did not receive local treatments, fluorouracil-based doublet chemotherapy combined with targeted agents has achieved progression-free survival (PFS) of up to 14 months and overall survival (OS) of up to 40 months, primarily in palliative settings [15,16]. However, despite advancements in systemic therapies, these treatments remain insufficient for achieving curative outcomes, underscoring the need for integrated approaches combining systemic therapy with local interventions. Among suitable patients, the combination of surgical resection with systemic therapy has demonstrated significant survival benefits, with 5-year survival rates reaching 30–35% in patients undergoing surgery, compared to 20% in those receiving only systemic therapy [17,18]. In a study by Creasy et al., 20% of patients with liver-limited mCRC who underwent surgical intervention achieved long-term cure following a 10-year follow-up [19]. Although the patients in that study underwent upfront surgical resection, their findings reinforce the importance of incorporating local treatment strategies into the management of potentially resectable liver mCRC. In our study, PFS and OS outcomes were consistent with previously reported literature, further supporting the role of conversion surgery in appropriately selected patients.

To date, no prospective studies have evaluated the impact of adding biological therapy on treatment outcomes in potentially resectable isolated liver metastatic colorectal cancer (mCRC). The only study evaluating the efficacy of induction chemotherapy is the NEW EPOC trial. In this study, patients with resectable isolated liver metastatic colorectal cancer were assessed, and the addition of anti-EGFR therapy was found to have a detrimental effect [20]. While induction chemotherapy has been associated with numerically improved objective response rates, this enhancement has not consistently translated into a survival benefit.

Our study was not designed to assess resectability rates, as it specifically only included patients who had undergone resection for isolated liver mCRC. Consequently, the high objective response rates observed reflect the fact that these patients had already been selected for surgery. These findings suggest that appropriate treatment selection can achieve effective micrometastatic disease control in patients with resected liver-limited mCRC.

Among patients with RAS/RAF wild-type right-sided colon tumors, PFS was comparable between those treated with anti-EGFR plus doublet chemotherapy and those receiving anti-VEGF plus doublet therapy. Although the addition of anti-EGFR therapy resulted in a numerical OS benefit over anti-VEGF, this difference did not reach statistical significance. Previous landmark studies, including FIRE-3 [21] and CALGB/SWOG 80405 [22], demonstrated that incorporating biological therapies into fluorouracil-based treatment provided a survival advantage in RAS wild-type mCRC. The FIRE-3 trial specifically showed a survival benefit with anti-EGFR therapy compared to anti-VEGF, while the CALGB/SWOG 80,405 study did not confirm this finding. The Phase 2 PEAK trial [23] further demonstrated an OS advantage with anti-EGFR therapy in RAS wild-type patients. Post hoc analyses of the PEAK [23] and PRIME [24] trials indicated that anti-EGFR therapy particularly benefits patients with left-sided colon tumors and RAS wild-type status [25].

In our study, the addition of anti-EGFR therapy to doublet chemotherapy resulted in numerically longer PFS and OS compared to anti-VEGF therapy in RAS/RAF wild-type right-sided colon tumors. Notably, prior studies investigating anti-EGFR therapy were conducted in patients with metastatic disease who did not undergo curative surgery. The PARADIGM study [26] revealed that more genetic alterations, including RTK/RAS mutations, emerged during disease progression in patients receiving anti-EGFR therapy compared to those treated with anti-VEGF therapy. Based on these findings, we hypothesize that implementing a curative surgical approach before the accumulation of these genetic alterations, potentially linked to anti-EGFR resistance, may further improve PFS in RAS/RAF wild-type mCRC. In patients with RAS/RAF-mutant right-sided colon tumors, escalation from doublet therapy plus bevacizumab to triplet therapy plus bevacizumab did not confer a PFS advantage. Previous studies have suggested that triplet therapy plus bevacizumab provides modest improvements in overall response rate (ORR) and PFS in advanced-stage right-sided colon tumors [27,28]. However, in our study, this benefit was not observed in patients who underwent conversion surgery. Furthermore, the addition of bevacizumab to doublet therapy did not result in an OS advantage in RAS/RAF-mutant right-sided colon cancer. A potential explanation for these findings is the relatively small number of patients receiving bevacizumab in combination with triplet therapy in our cohort. The CAIRO-5 study demonstrated that triplet-based therapy combined with bevacizumab was associated with longer PFS in both right-sided colon cancers and RAS/RAF-mutant left-sided colon cancers (10.6 vs. 9.0 months, *p* = 0.032) [28]. However, a key distinction in CAIRO-5 was the higher rate of local treatment in patients receiving triplet therapy (51% vs. 37%, *p* = 0.013), which could have contributed to the observed PFS advantage. In contrast, all patients underwent conversion surgery in our study, potentially explaining why a similar PFS benefit was not observed. In patients with RAS/RAF wild-type left-sided colon tumors, anti-EGFR plus doublet therapy was associated with numerically superior PFS and OS compared to anti-VEGF plus doublet therapy, though this difference did not reach statistical significance. Meanwhile, in RAS/RAF-mutant left-sided colon tumors, no significant differences in PFS or OS were observed among doublet therapy, doublet plus bevacizumab, or triplet-based therapy. These findings suggest that while anti-EGFR therapy remains a promising option for RAS/RAF wild-type left-sided colon tumors, further studies with larger cohorts are needed to confirm its survival benefit.

Circulating tumor DNA (ctDNA) studies have emerged as a crucial tool in cancer research, providing insights into minimal residual disease and treatment response [29,30]. In the Japan GALAXY study, which evaluated stage 4 colorectal cancer patients who underwent surgery, the presence of post-operative ctDNA and failure to achieve ctDNA clearance were strongly associated with disease recurrence [29]. This study highlights the importance of post-operative ctDNA monitoring in guiding adjuvant chemotherapy decisions. However, the impact of induction chemotherapy with or without biological agents on achieving ctDNA clearance remains an area of ongoing investigation, necessitating further research to optimize treatment strategies.

The median time from diagnosis to surgery was 6 months, and PFS and OS were comparable between patients who underwent surgery before or after 6 months of systemic therapy. These findings suggest that resectability can be reassessed throughout the treatment period, as patients who exhibit a delayed or deepening response to therapy may become surgical candidates at later stages. This highlights the feasibility of evaluating resectability at multiple time points during systemic therapy. However, it is important to note that these results apply only to patients who ultimately underwent surgery, and do not account for those who experienced disease progression during treatment and were not operated on. Patient selection played a crucial role in our study, as multiple factors influenced both PFS and OS. Even in cases where R0 resection was achieved, advanced T stage and the presence of lymph node metastases were associated with shorter PFS, a finding consistent with prior studies [31]. The correlation between higher T stage, nodal involvement, and poorer survival outcomes may be attributed to the increased risk of distant metastatic spread in these patients [32,33,34]. Additionally, among those initially deemed unresectable, patients with more than three liver metastases had significantly shorter PFS, aligning with previous reports by Iwatsuki et al. [35] and Okano et al. [36], which demonstrated that a higher metastatic burden negatively impacts survival outcomes.

In our study, overall survival (OS) was significantly shorter in patients who failed to achieve an objective response and in those with advanced T stage, emphasizing the importance of tumor response and disease burden in determining survival outcomes. Achieving an objective response not only increases the probability of R0 resection but also correlates with improved long-term survival. Indeed, patients in our cohort who exhibited an objective response to induction chemotherapy demonstrated superior survival outcomes. However, the lack of a statistically significant difference between induction therapy regimens may be attributed to variability in conversion surgery rates across different treatment groups. Since patients initially deemed unresectable underwent surgery at varying frequencies, selection bias within the study cohort may have influenced OS and PFS outcomes. Additionally, failure to achieve an objective response may serve as a surrogate marker for more aggressive tumor biology, further contributing to poorer survival outcomes.

Interestingly, a subset of patients in our study underwent conversion surgery despite achieving only a stable response to induction chemotherapy. Findings from the CAIRO-5 trial [28] suggest that the consensus rate among surgeons regarding operability decreased from 60% at baseline to 42% after chemotherapy, highlighting the dynamic nature of resectability assessment during systemic therapy. This reinforces the necessity of ongoing reassessment of surgical candidacy throughout treatment, particularly for patients demonstrating delayed or deepening responses. The lack of a standardized consensus in determining conversion surgery eligibility further underscores the need for more reliable predictive markers to guide surgical decision-making in this setting.

In our study, 16% of patients experienced grade 3/4 adverse events, with a higher incidence observed in the anti-EGFR therapy group. The most frequently reported toxicities were hematologic adverse events, including neutropenia, thrombocytopenia, and anemia, followed by gastrointestinal and skin-related toxicities. The incidence and nature of adverse events in our cohort were consistent with previously published data [28], emphasizing the importance of toxicity monitoring and patient selection when incorporating biological agents into induction chemotherapy regimens.

This study is retrospective in nature. Although operability decisions were made by a multidisciplinary team, the multicenter design resulted in a lack of universal consensus on surgical decision-making. Only patients who were eligible for R0 resection underwent surgery, while those treated with ablation, embolization, or multimodal approaches were excluded. Another limitation was the variability in surgical sequencing, as both primary tumor resection and liver metastasectomy were performed either in the same session or sequentially, with liver-first surgery being the more common approach. However, a key constraint was the absence of data regarding the interval between liver metastasectomy and primary tumor resection, particularly for patients undergoing liver-first procedures. Furthermore, the small sample sizes in certain subgroups limited the statistical power of some analyses. Larger, prospective studies are needed to validate our findings, refine optimal treatment strategies, and better define the impact of induction chemotherapy and surgical sequencing in conversion therapy for isolated liver mCRC.

## 5. Conclusions

Our findings reinforce the critical role of surgical intervention in improving long-term survival outcomes for patients with potentially resectable liver metastatic colorectal cancer (mCRC). In this study, progression-free survival (PFS) and overall survival (OS) outcomes were evaluated based on tumor localization, RAS/RAF mutation status, and systemic therapy regimens, contributing valuable insights into treatment optimization in the conversion setting.

For patients with RAS/RAF wild-type mCRC, PFS and OS were comparable between those treated with anti-EGFR therapy and anti-VEGF therapy, suggesting that both targeted therapies remain viable options in this subgroup. In RAS/RAF mutant right-sided colon cancer, anti-VEGF plus triplet therapy did not provide a significant survival benefit over anti-VEGF plus doublet therapy. Similarly, in RAS/RAF mutant left-sided colon cancer, no significant differences were observed in PFS or OS among doublet therapy, triplet therapy, or doublet plus anti-VEGF therapy, suggesting that treatment intensification strategies should be carefully individualized based on patient-specific factors rather than standard escalation.

This study also highlights the importance of reassessing resectability throughout treatment, as some patients who initially exhibited only stable disease still achieved conversion surgery. This reinforces the need for ongoing multidisciplinary evaluation, particularly given the evolving nature of tumor biology and treatment response over time. Furthermore, the lack of survival difference between shorter and longer durations of systemic therapy prior to surgery suggests that patients should not be excluded from resectability reassessment solely based on early imaging responses, as deeper responses may develop with extended treatment.

Given the heterogeneity of treatment responses and the complex decision-making process surrounding resectability, larger, prospective studies are warranted to further define optimal induction therapy strategies based on tumor localization, mutation status, and molecular response markers. A better understanding of predictive biomarkers, including ctDNA dynamics, will be crucial in guiding treatment selection, optimizing conversion rates, and ultimately improving long-term outcomes for patients with potentially resectable liver mCRC.

## Figures and Tables

**Table 1:** Patients and tumor characteristics.

Variables		Whole Group (*n* = 116)	Right Colon (*n* = 28)	Left Colon (*n* = 40)	Rectum (*n* = 48)	*p*
Gender	Female	44 (38%)	11	19	14	0.208
	Male	72 (62%)	17	21	34	
Age		57	59	57	55	0.229
ECOG PS	0	61 (52%)	15	18	28	0.532
	1	52 (45%)	13	20	19	
	2	3 (3%)	0	2	1	
Metastases in the same segment	Yes	56 (48%)	15	13	28	0.044
	No	60 (52%)	13	27	20	
Number of Liver metastases	≤3	84 (72%)	21	28	35	0.897
	>3	32 (28%)	7	12	13	
Largest Metastasis Diameter (mm)		38	46	38	36	0.484
Chemotherapy	Doublet	14 (13%)	3	7	4	0.529
	Anti-VEGF Group	56 (52%)	15	16	25	
	Anti-EGFR Group	37 (35%)	7	15	15	
Chemotherapy Regimes	Capox	1 (1%)	0	0	1	0.689
	Folfox	13 (11%)	3	7	3	
	Folfox + Anti-EGFR	25 (22%)	6	9	10	
	Folfox + Anti-VEGF	33 (28%)	8	12	13	
	Folfiri + Anti-EGFR	12 (10%)	1	6	5	
	Folfiri + Anti-VEGF	11 (10%)	5	1	5	
	Capox+ Anti-VEGF	12 (10%)	2	3	7	
	Folfoxiri	1 (1%)	0	1	0	
	Folfoxiri + Anti-EGFR	1 (1%)	0	0	1	
	Folfoxiri+ Anti-VEGF	7 (6%)	3	1	3	
Number Of Chemotherapy Cycles Administered		8	9	9	8	0.462
Grade 3/4 AE	Absence	97 (84%)	25	32	40	0.594
	Presence	19 (16%)	3	8	8	
Radiographic response	CR	24 (21%)	4	6	14	0.268
	PR	83 (71%)	21	29	33	
	SD	8 (7%)	3	4	1	
	PD	1 (1%)	0	1	0	
ypT	T1	7 (6%)	1	1	5	0.543
	T2	15 (13%)	5	2	7	
	T3	70 (60%)	17	26	27	
	T4	24 (21%)	5	10	9	
ypN	N0	40 (35%)	7	14		0.569
	N1	54 (47%)	15	19		
	N2	21 (18%)	5	7		
	N3	1 (1%)	1	0		
Time to Surgery (Months) Median, IQR		6.0	7.2	9.8	9.2	0.446
Number of lymph nodes removed	<12	44 (38%)	8	14	22	0.292
	≥12	72 (62%)	20	26	26	
Grade	N/A	37 (32%)	8	16	13	0.743
	Grade1	29 (25%)	7	9	13	
	Grade2	32 (28%)	10	7	15	
	Grade3	16 (14%)	3	7	6	
	Grade4	2 (1%)	0	1	1	
Differentiation	N/A	19 (16%)	6	7	6	0.831
	Good	35 (30%)	7	11	17	
	Intermediate	54 (47%)	14	18	22	
	Poor	8 (7%)	1	4	3	
Tumor Regression Score	Grade 0	1 (1%)	0	1	0	0.488
	Grade 1	7 (6%)	4	2	1	
	Grade 2	42 (36%)	9	16	17	
	Grade 3	38 (33%)	8	12	18	
	N/A	28 (24%)	7	9	12	
Resection	N/A	14 (12%)	3	2	9	0.227
	R0	88 (76%)	22	32	34	
	R1	11 (9%)	2	6	3	
	R2	3 (3%)	1	0	2	
KRAS	Wild	65 (56%)	13	25	27	0.421
	Mutant	51 (44%)	15	15	21	
NRAS	Wild	111 (96%)	28	39	44	0.290
	Mutant	5 (4%)	0	1	4	
BRAF	Wild	103 (89%)	27	36	40	0.465
	Mutant	4 (3%)	0	1	3	
	N/A	9 (8%)	1	3	5	
MSI	MSS	79 (99%)	16	29	34	0.153
	MSI-H	1 (1%)	1	0	0	
Radiotherapy (for rectum adenocarcinoma)	Received	16 (57%)	0	0	16	
	Not Received	12 (43%)	28	40	12	
Adjuvant Chemotherapy	Received	38 (72%)				
	Not Received	15 (28%)				
Recurrence	Presence	86 (74%)				
Chemotherapy after recurrence	Administered	84 (98%)				

This table summarizes key patient characteristics, including age, gender, ECOG performance status (PS), and primary tumor location (right colon, left colon, rectum). The size and number of liver metastases, chemotherapy cycles, and time to surgery are presented. Chemotherapy regimens (doublet, doublet + anti-EGFR, doublet + anti-VEGF) and the frequency of grade 3/4 adverse events (AEs) are also reported. Radiographic responses are categorized as complete response (CR), partial response (PR), stable disease (SD), or progressive disease (PD). Pathologic staging includes tumor (ypT) and nodal (ypN) classification after chemotherapy. Surgical outcomes include the number of lymph nodes removed, perineural invasion (PNI), lymphovascular invasion (LVI), tumor grade, differentiation, tumor regression score, and resection margins (R0, R1, R2). Molecular characteristics such as KRAS, NRAS, BRAF mutations, and microsatellite instability (MSI) status are also detailed, along with data on radiotherapy, adjuvant chemotherapy, recurrence rates, and post-recurrence treatment.

**Table 2:** Univariate and Multivariate Analysis Results on Progression-Free Survival.

	Univariate Analyses		Multivariate Analyses	
	HR (95% Cl)	*p*	HR (95% CI)	*p*
Age		0.339		
<65	1			
≥65	0.78 (0.47–1.31)			
Gender		0.590		
Female	1			
Male	1.13 (0.72–1.77)			
ECOG		0.181		
0	1			
1	1.35 (0.87–2.07)			
Localization		0.988		
Right colon	1			
Left colon	0.96 (0.55–1.68)			
Rectum	0.96 (0.55–1.67)			
KRAS Mutation		0.810		
No	1			
Yes	1.05 (0.69–1.61)			
NRAS Mutation		0.352		
No	1			
Yes	1.58 (0.64–3.93)			
BRAF Mutation		0.480		
No	1			
Yes	0.68 (0.21–2.15)			
Presence of RAS/RAF mutation		0.574		
No	1			
Yes	1.13 (0.74–1.72)			
Microsatellite Status		0.517		
MSS	1			
MSI-H	2.09 (0.28–15.40)			
pT stage		0.018		0.072
ypT1-T2	1		1	
ypT3-T4	2.02 (1.07–3.82)		2.33	
pN stage		0.109		0.053
ypN0	1		1	
ypN+	1.43 (0.92–2.24)		1.74	
Grade		0.259		
1–2	1			
3–4	1.41 (0.79–2.53)			
Differentiation		0.626		
Good and Int	1			
Poor	0.82 (0.35–1.89)			
Tumor Regression Score		0.066		
0–1	1			
2–3	1.57 (0.97–2.52)			
Presence of metastases in One Liver Segment		0.005		
Yes	1			
No	1.91 (1.21–3.00)			
Number of Metastases		0.007		0.002
≤3	1		1	
>3	2.01 (1.24–3.28)		2.65	
Largest diameter of Liver metastases		0.374		
<30 mm	1			
≥30 mm	1.22 (0.79–1.86)			
Objective Response		0.229		
Absence	1			
Presence	1.67 (0.77–3.63)			
Time to surgery		0.116		
<6 months	1			
≥6 months	1.41 (0.92–2.17)			
Chemotherapy		0.053		
Doublet	1		1	
Doublet + Anti-EGFR	0.97 (0.44–2.13)		1.04	0.927
Doublet + Anti-VEGF	1.84 (0.90–3.77)		2.22	0.056
Radiotherapy		0.196		
Not Received	1			
Received	0.58 (0.25–1.32)			
Adjuvant Chemotherapy		0.934		
Not Received	1			
Received	0.97 (0.50–1.88)			

This table presents the univariate and multivariate analysis of factors influencing PFS. Variables include demographic factors (age, gender), ECOG performance status, tumor location, mutation status (KRAS, NRAS, BRAF, RAS/RAF), microsatellite status (MSS/MSI-H), pathologic staging (ypT, ypN), presence of perineural invasion (PNI) and lymphovascular invasion (LVI), tumor grade and differentiation, tumor regression score, and the number and location of liver metastases. The analysis also examines the impact of chemotherapy regimens, radiotherapy, adjuvant chemotherapy, and the time to surgery on PFS. Hazard ratios (HR), 95% confidence intervals (CI), and *p*-values are reported to highlight the significance of each variable.

**Table 3:** Univariate and Multivariate Analysis Results on Overall Survival.

	Univariate Analyses		Multivariate Analyses	
	HR (95% Cl)	*p*	HR (95% CI)	*p*
Age		0.587		
<65	1			
≥65	0.83 (0.42–1.66)			
Gender		0.852		
Female	1			
Male	0.95 (0.54–1.65)			
ECOG		0.873		
0	1			
1	0.96 (0.55–1.66)			
Localization		0.834		
Right colon	1			
Left colon	0.88 (0.44–1.77)			
Rectum	0.81 (0.41–1.60)			
KRAS Mutation		0.398		
No	1			
Yes	1.27 (0.74–2.18)			
NRAS Mutation		0.793		
No	1			
Yes	0.83 (0.20–3.42)			
BRAF Mutation		0.043		
No	1			
Yes	0.46 (0–21.85)			
Presence of RAS/RAF mutation		0.551		
No	1			
Yes	1.18 (0.69–2.03)			
Microsatellite Status		0.407		
MSS	1			
MSI-H	0.05 (0–125.044)			
pT stage		0.014		0.040
ypT1-T2	1		1	
ypT3-T4	2.45 (1.10–5.44)		12.2 (1.45–16.87)	
pN stage		0.391		
ypN0	1			
ypN+	1.28 (0.73–2.23)			
Perineural Invasion		0.264		
No	1			
Yes	1.42 (0.77–2.61)			
Lymphovascular Invasion		0.625		
No	1			
Yes	0.86 (0.47–1.58)			
Grade		0.044		
1–2	1			
3–4	2.14 (1.05–4.36)			
Differentiation		0.162		
Good and Intermediate	1			
Poor	1.95 (0.82–4.62)			
Tumor Regression Score		0.015		
0–1	1			
2–3	2.18 (1.17–4.08)			
Presence metastases in One Liver Segment		0.075		
No	1			
Yes	1.66 (0.94–2.91)			
Number of Metastases		0.181		
≤3	1			
>3	1.54 (0.83–2.84)			
Largest diameter of liver metastases		0.897		
<30 mm	1			
≥30 mm	1.04 (0.60–1.80)			
Objective Response		0.042		0.023
Absence	1		1	
Presence	0.36 (0.16–0.86)		0.12 (0.08–0.74)	
Time to surgery		0.134		
<6 months	1			
≥6 months	1.52 (0.88–2.62)			
Chemotherapy		0.231		
Doublet	1			
Doublet + Anti-EGFR	0.87 (0.35–2.18)			
Doublet + Anti-VEGF	1.53 (0.67–3.49)			
Radiotherapy		0.164		
Not Received	1			
Received	0.49 (0.18–1.33)			
Adjuvant Chemotherapy		0.682		
Not Received	1			
Received	1.19 (0.51–2.80)			

This table presents the univariate and multivariate analysis of factors affecting OS. Variables include age, gender, ECOG performance status (PS), tumor location, mutation status (KRAS, NRAS, BRAF, RAS/RAF), microsatellite status (MSS/MSI-H), pathologic staging (ypT, ypN), perineural invasion (PNI), lymphovascular invasion (LVI), tumor grade, differentiation, tumor regression score, and the number and location of liver metastases. The analysis also evaluates the impact of chemotherapy regimens, radiotherapy, adjuvant chemotherapy, and objective response on OS. Hazard ratios (HRs), 95% confidence intervals (CI), and *p*-values are reported to indicate the significance of each factor.

## Data Availability

The original contributions presented in this study are included in the article/Supplementary Materials. Further inquiries can be directed to the corresponding author(s).

## Supplementary Materials

The following supporting information can be downloaded at: https://www.mdpi.com/article/10.3390/medicina61050776/s1.
