# Supplementary material for: Long-Term Outcomes of Patients Undergoing Conversion Surgery After Induction Chemotherapy: Turkish Oncology Group Study
Source: Medicina (Kaunas). 2025 Apr 22;61(5):776. doi: 10.3390/medicina61050776 (PMC12113550; doi:10.3390/medicina61050776)
Supplement: Supplementary file 1 [file medicina-61-00776-s001.zip › medicina-3541402-supplementary.pdf]

Supplementary

Supplementary Table S1. Patient and tumor characteristics according to chemotherapy.

		Chemotherapy			
		Doublet	Doublet +VEGF	Doublet +EGFR	p
Gender	Female	10	19	12	**0.024**
	Male	4	37	25	
Age		58	56	58	0.476
ECOG PS	0	8	31	17	0.743
	1	6	24	18	
Metastases in the same segment	Yes	6	25	20	0.625
	No	8	31	17	
Number of Liver metastases	≤3	11	38	27	0.695
	>3	3	18	10	
Largest metastasis diameter (mm)		25.9	43.9	38.5	0.226
Number of chemotherapies administered		7	8	11	**0.004**
Grade 3/4 AE	Absence	13	52	23	**<0.001**
	Presence	1	4	14	
Radiographic response	CR	2	13	9	0.374
	PR	12	35	27	
	SD	0	7	1	
	PD	0	1	0	
ypT	T1	0	3	4	0.214
	T2	2	6	6	
	T3	8	33	25	
	T4	4	14	2	
ypN	N0	6	23	10	0.246
	N1	8	22	16	
	N2	0	10	11	
	N3	0	1	0	
Time to Surgery (month)		7.1	9.8	7.5	0.328
Number of lymph node removed	<12	5	22	15	0.952
	≥12	9	34	22	
Grad	N/A	6	18	10	0.107
	Grade1	7	13	8	
	Grade2	0	14	15	
	Grade3	1	10	3	
	Grade4	0	1	1	
Differentiation	N/A	0	10	7	0.119
	Good	8	11	11	
	Intermediate	6	30	17	
	Poor	0	5	2	
Tumor Regression Score	Grade 0	0	0	1	0.463
	Grade 1	0	5	2	
	Grade 2	8	16	14	
	Grade 3	3	24	9	
	N/A	3	11	11	
Resection	N/A	0	6	7	0.303
	R0	13	43	24	
	R1	1	5	5	
	R2	0	2	1	

KRAS	Wild	5	21	37	**<0.001**
	Mutant	9	35	0	
NRAS	Wild	13	52	37	0.250
	Mutant	1	4	0	
BRAF	Wild	13	50	33	0.800
	Mutant	1	2	1	
	N/A	0	4	3	
MSI	MSS	12	36	26	0.594
	MSI-H	0	1	0	

This continuation of Supplementary Table 3 compares additional patient and tumor characteristics based on chemotherapy regimens (doublet therapy, doublet therapy plus anti-VEGF, doublet therapy plus anti-EGFR. Variables include gender, Eastern Cooperative Oncology Group (ECOG) performance status (PS), presence of liver metastases in the same segment, number of liver metastases, grade 3/4 adverse events (AEs), radiographic response (complete response [CR], partial response [PR], stable disease [SD], progressive disease [PD]), pathologic staging (ypT, ypN), number of lymph nodes removed, presence of perineural invasion (PNI), presence of lymphovascular invasion (LVI), tumor grade, tumor differentiation, tumor regression score, resection status (R0, R1, R2), and molecular characteristics (KRAS, NRAS, BRAF mutations, and microsatellite instability [MSI] or microsatellite stable [MSS]). Chi-square tests (χ^2) and p-values are used to assess statistical significance across the groups.

Supplementary Table S2: Chemotherapies given according to tumor localization and mutation status

	**FOLFOX/ FOLFIRI/ CAPOX n (%)**	**FOLFOX/ FOLFIRI/ CAPOX + CETUXİMAB/ PANİTİMUMAB n (%)**	**FOLFOX/ FOLFIRI/ CAPOX + BEVACİZUMAB n (%)**	**FOLFOXIRI n (%)**	**FOLFOXIRI + CETUXİMAB/ PANİTİMUMAB n (%)**	**FOLFOXIRI + BEVACİZUMAB n (%)**
**Right Colon**	3 (10)	7 (25)	15 (54)	0 (0)	0 (0)	3 (11)
RAS/RAF wild	0 (0)	7 (54)	5 (38)	0 (0)	0 (0)	1 (8)
RAS/RAF mutation	3 (20)	0 (0)	10 (67)	0 (0)	0 (0)	2 (13)
**Left Colon**	7 (18)	15 (38)	16 (40)	1 (2)	0 (0)	1 (2)
RAS/RAF wild	4 (17)	15 (63)	5 (20)	0 (0)	0 (0)	0 (0)
RAS/RAF mutation	3 (19)	0 (0)	11 (69)	1 (6)	0 (0)	1 (6)
**Rectum**	4 (8)	15 (31)	25 (52)	0 (0)	1 (3)	3 (6)
RAS/RAF wild	0 (0)	15 (60)	9 (36)	0 (0)	1 (4)	0 (0)
RAS/RAF mutation	4 (17)	0 (0)	16 (70)	0 (0)	0 (0)	3 (13)

This table presents the distribution of chemotherapy regimens administered to patients based on tumor location (right colon, left colon, rectum) and RAS/RAF mutation status (wild-type or mutant). Chemotherapy regimens include FOLFOX, FOLFIRI, CAPOX, and combinations with cetuximab, panitumumab, or bevacizumab. The number and percentage of patients receiving each regimen are detailed for both wild-type and mutant RAS/RAF groups across tumor locations.

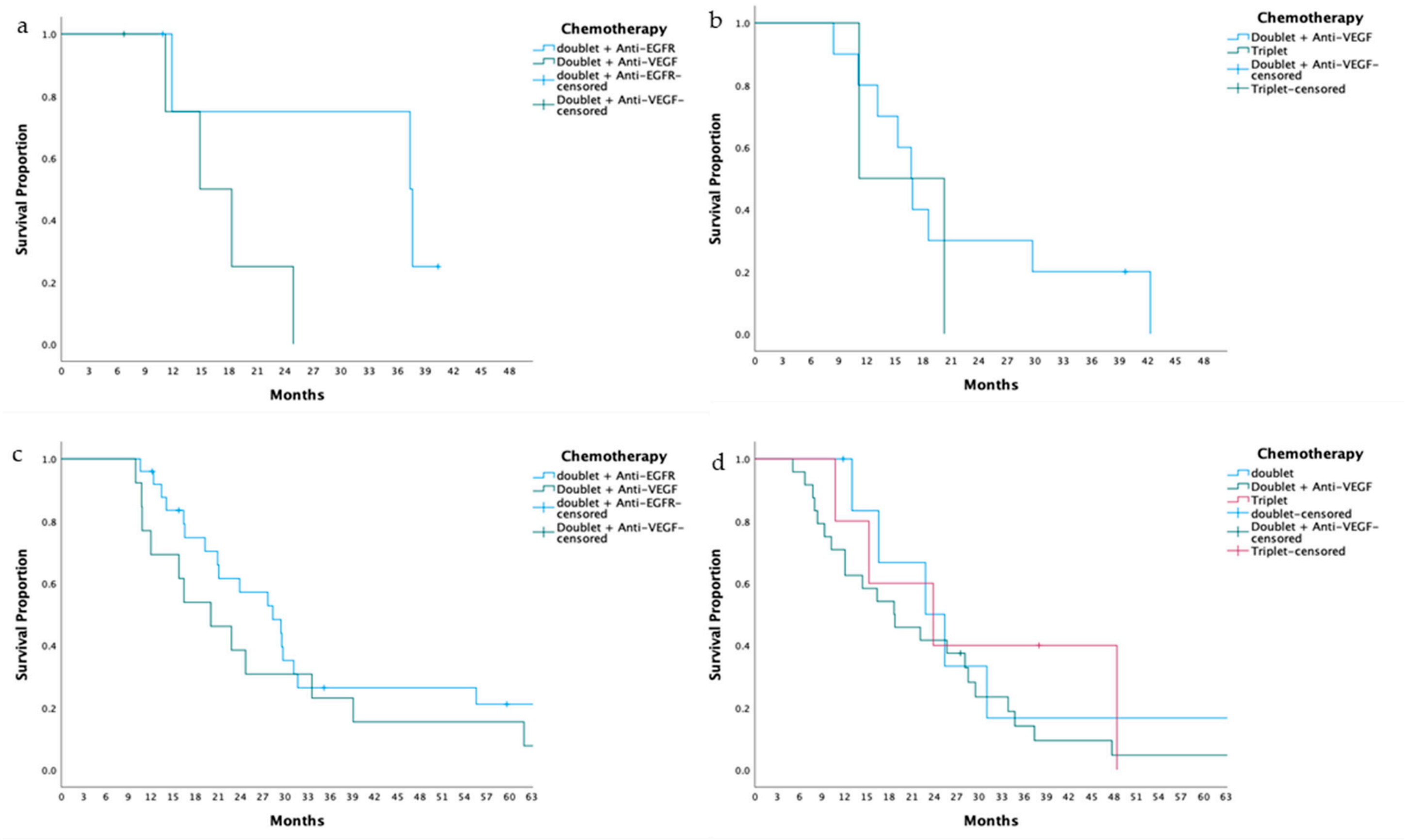

Supplementary Figure S1: Progression-Free Survival (PFS) by Therapy and Mutation Status

(a) RAS/RAF wild-type right colon tumors: Comparison of doublet therapy plus anti-EGFR vs. doublet therapy plus anti-VEGF (37.6 vs. 15.0 months, p=0.059). (b) RAS/RAF mutant right colon tumors: Comparison of doublet therapy plus anti-VEGF vs. triplet therapy plus anti-VEGF (16.7 vs. 11.2 months, p=0.246). (c) RAS/RAF wild-type left colon tumors: Comparison of doublet therapy plus anti-EGFR vs. doublet therapy plus anti-VEGF (28.3 vs. 20.0 months, p=0.173). (d) RAS/RAF mutant left colon tumors: Comparison of doublet therapy vs. triplet therapy vs. doublet therapy plus anti-VEGF (22.8 vs. 23.9 vs. 18.6 months, p=0.501).

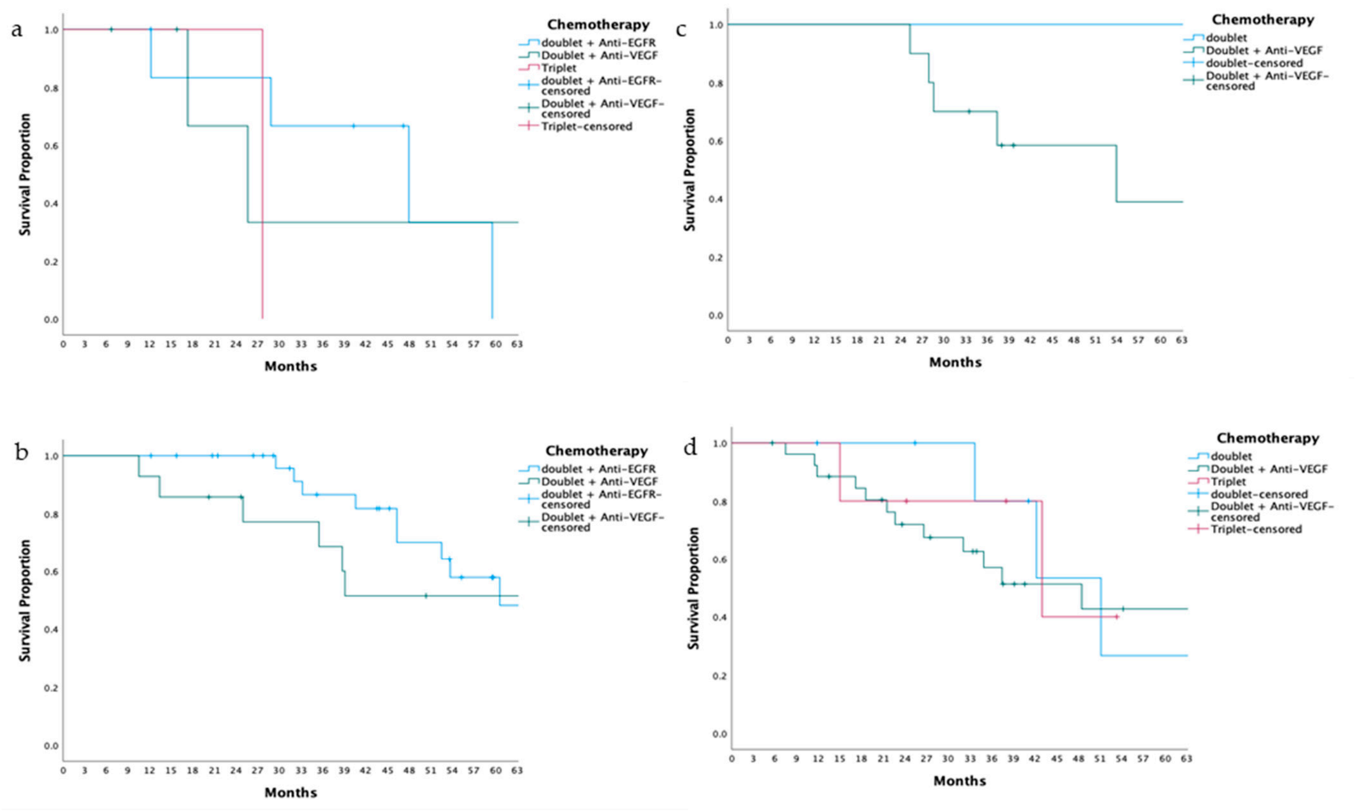

Supplementary Figure S2: Overall Survival (OS) by Therapy and Mutation Status

(a) RAS/RAF wild-type right colon tumors: Comparison of doublet therapy plus anti-EGFR vs. doublet therapy plus anti-VEGF vs. triplet therapy (48.0 vs. 25.6 vs. 27.7 months, p=0.711). (b) RAS/RAF mutant right colon tumors: Comparison of doublet therapy plus anti-VEGF vs. doublet therapy alone (not estimable vs. 53.9 months, p=0.172). (c) RAS/RAF wild-type left colon tumors: Comparison of doublet therapy plus anti-EGFR vs. doublet therapy plus anti-VEGF (60.6 vs. 69.0 months, p=0.230). (d) RAS/RAF mutant left colon tumors: Comparison of doublet therapy vs. triplet therapy vs. doublet therapy plus anti-VEGF (51.1 vs. 43.0 vs. 48.5 months, p=0.798).
