# Supplementary material for: Long-Term Outcomes of Patients Undergoing Conversion Surgery After Induction Chemotherapy: Turkish Oncology Group Study
Source: Medicina (Kaunas). 2025 Apr 22;61(5):776. doi: 10.3390/medicina61050776 (PMC12113550; doi:10.3390/medicina61050776)
Supplement: Supplementary file 1 [file medicina-61-00776-s001.zip › patient number.pdf]

Supplementary Table S3: Patients number from each center.

**Hospital**	**Number of Patients**
Ankara Bilkent City Hospital	14
UHS İzmir University Hospital	5
Ankara Batıkent Medical Park Hospital	7
A.Y. Ankara Oncology Training and Research Hospital	12
Dokuz Eylül University Hospital	21
Adana City Hospital	5
Sakarya Training and Research Hospital	5
Pamukkale University Hospital	11
Bezmialem University Hospital	15
Mersin City Hospital	15
Ege University Hospital	6
